# Enhanced antibacterial activity and superior biocompatibility of cobalt-deposited titanium discs for possible use in implant dentistry

**DOI:** 10.1016/j.isci.2024.108827

**Published:** 2024-01-09

**Authors:** Vaibhav Madiwal, Bhushan Khairnar, Jyutika Rajwade

**Affiliations:** 1 Nanobioscience Group, Agharkar Research Institute, G. G. Agarkar Road, Pune, Maharashtra 411 004, India; 2 Savitribai Phule Pune University, Homi Bhabha Road, Pune, Maharashtra 411 007, India

**Keywords:** Dentistry, Materials science, Biomaterials, Biomedical materials

## Abstract

The clinical success of implants depends on rapid osseointegration, and new materials are being developed considering the increasing demand. Considering cobalt (Co) antibacterial characteristics, we developed Co-deposited titanium (Ti) using direct current (DC) sputtering and investigated it as a new material for implant dentistry. The material was characterized using atomic absorption spectroscopy, scanning electron microscopy-energy dispersive X-ray spectroscopy, and X-ray photoelectron spectroscopy. The material’s surface topography, roughness, surface wettability, and hardness were also analyzed. The Co thin film (Ti-Co_15_) showed excellent antibacterial effects against microbes implicated in peri-implantitis. Furthermore, Ti-Co_15_ was compatible and favored the attachment and spreading of MG-63 cells. The alkaline phosphatase and calcium mineralization activities of MG-63 cells cultured on Ti-Co_15_ remained unaltered compared to Ti. These data correlated well with the time-dependent expression of *ALP, RUNX-2,* and *BMP-2* genes involved in osteogenesis. The results demonstrate that Co-deposited Ti could be a promising material in implant dentistry.

## Highlights

• Cobalt surface coating was developed using sputtering for implant dentistry application
• Co deposition on Ti (Ti-Co_15_) increased hydrophilicity and surface free energy
• Ti-Co_15_ exhibited antibacterial activity against peri-implantitis-causing microbes
• Ti-Co_15_ surface promoted osteoblasts attachment, spreading, and mineralization

## Graphical abstract

**Figure:**
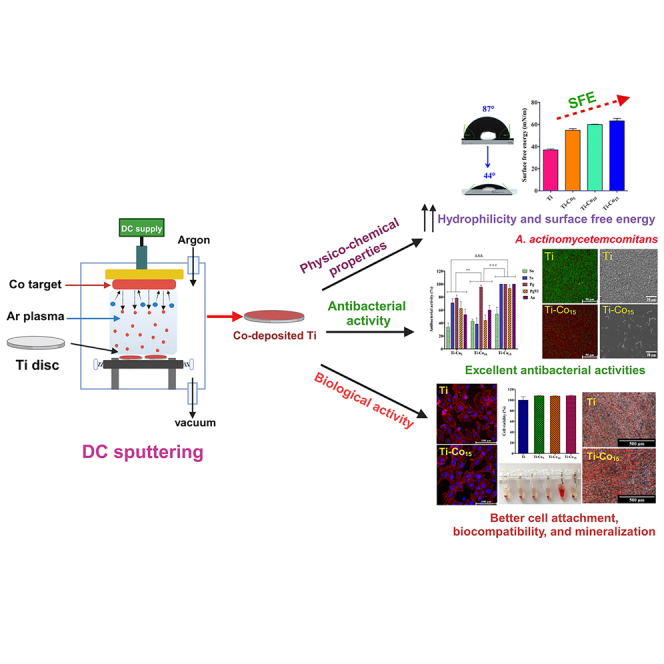

## Introduction

Loss of teeth affects the facial appearance and poses serious issues affecting the individual’s health, psychological, and behavioral aspects. To overcome these problems, advanced prosthetic dentistry offers treatment modalities such as dental implants, bridges, and dentures.^1^ According to the American Dental Association, around 5 million implants are surgically placed each year, with an increase of ∼9.1%.^2^ In modern dentistry, Ti and its alloys are widely used for manufacturing dental implants, primarily due to their excellent mechanical properties, corrosion resistance, and biocompatibility.^3^^,^^4^ However, Ti implants lack antimicrobial activity, and the screw-like design favors bacterial colonization,^5^ which occurs immediately after implant surgery. Research findings indicate that the adherence of salivary proteins to the implant surface facilitates the attachment of primary colonizers. The cell-to-cell attachment with secondary colonizers follows, forming a complex multi-species biofilm on implant surfaces.^6^^,^^7^ Biofilm formation (also called dental plaque, responsible for the inflammation of soft connective tissue) and subsequent peri-implantitis (the progressive loss of bone around the implant), causes implant failures in about 5–10% cases.^8^^,^^9^

Peri-implant diseases have been predominantly associated with Gram-negative anaerobic microbiota such as *Aggregatibacter actinomycetemcomitans, Porphyromonas gingivalis, Bacteroides forsythus, Prevotella intermedia, Treponema denticola,* and so forth.^10^^,^^11^^,^^12^ Recent studies propose surface modifications of the implant to improve its antimicrobial and osteointegration properties.^13^^,^^14^ The surface topography, chemical composition, and surface free energy of Ti play a pivotal role in initial osteoblast attachment, proliferation, and mineralization of the extracellular matrix during the osseointegration process.^15^ To improve host cell attachment and osseointegration, procedures such as sand-blasting, acid-etching, anodization, laser treatment, UV-photofunctionalization, and plasma treatment were explored, which cause implant surface modification at the micro- and nanoscale.^16^^,^^17^ These techniques have proven to be effective in the initial osteoblast attachment, proliferation, and increased expression of ontogenesis-related genes, thereby improving the early osseointegration process.^18^^,^^19^^,^^20^ There is a race between bacteria and host cells for the attachment on the implant surface, and the antibacterial property of implant surfaces can certainly enhance early osseointegration, thus, ensuring the long-term survival of the implant.^21^ Implant surface modifications with silver (Ag), copper (Cu), and zinc (Zn) were demonstrated as an effective antimicrobial strategy.^22^^,^^23^^,^^24^^,^^25^ Amongst the techniques that impart surface modification with metals, the 'sputtering' technique offers precise control over the process parameters, resulting in the formation of a uniform thin film. The sputtering technique has been used in diverse fields, such as biosensors, surgical instruments, orthopedic implants, and fabrics/textiles.^26^^,^^27^^,^^28^ For the implant surfaces modified by metals, the antibacterial activity is governed by the metal ions released from the surface of the thin film, which cause physical damage to the membrane, inactivate cellular enzymes, and exert damaging effects on the genetic material, eventually leading to cell death.^29^^,^^30^ Due to the multi-level effects and broad-spectrum antibacterial activity of metals, researchers have focused on developing metallic surface coatings.

Co is an essential trace element and has been a constituent of implant alloys for over four decades.^31^ Recent studies indicate that Co has antibacterial activities.^32^^,^^33^^,^^34^ Similar to our previous study,^22^ we deposited a nanometer-thick layer of Co on Ti using DC sputtering and investigated it as a new material for implant dentistry. After fabrication, the physicochemical characterization was completed, and the antibacterial activity of the Co-deposited Ti was studied against periodontal pathogens. The *in vitro* biocompatibility of Co-deposited Ti was assessed using MG-63 cells.

## Results

### Surface morphology and topology analysis

**Figure 1:**
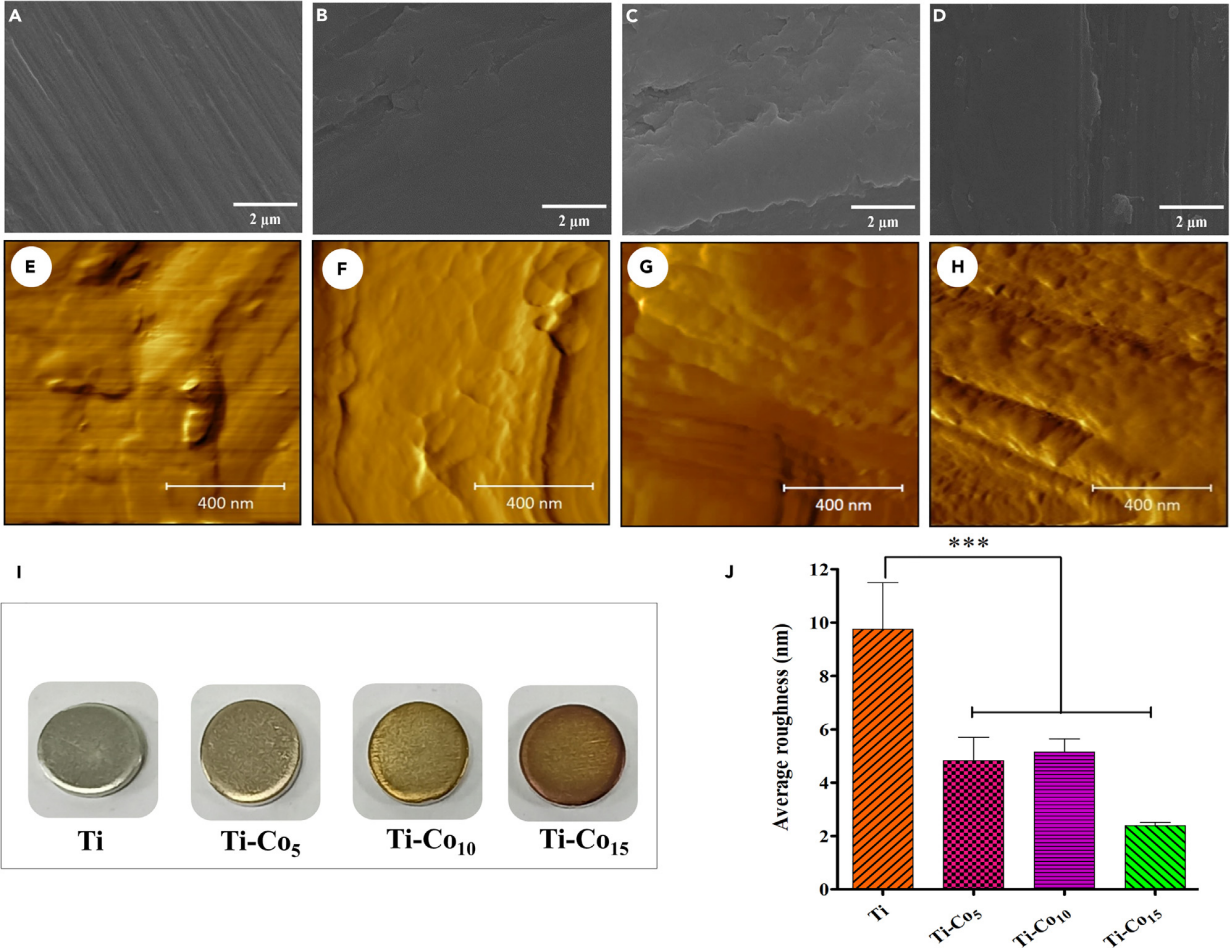
Scanning electron microscopy, atomic force microscopy and photographs of Ti and Co-deposited Ti discs Scanning electron microscopy images of Ti (A), Ti-Co_5_ (B), Ti-Co_10_ (C), and Ti-Co_15_ (D). Scale bar represents 2 μm. Atomic force microscopy images of Ti (E), Ti-Co_5_ (F), Ti-Co_10_ (G), and Ti-Co_15_ (H). Scale bar represents 400 nm. Photographic images of Ti and Co-deposited Ti discs (I). Average surface roughness of Ti and Co-deposited Ti discs (J). ∗∗∗ indicates p < 0.001 when compared to Ti.

The surface morphology of the control (Ti) and Co-deposited Ti samples was studied using SEM, as depicted in Figures 1A–1D. The Ti surface showed parallel surface scratches, probably generated during the polishing process (Figure 1A). Deposition of Co on the Ti discs resulted in a change in surface morphology compared to the Ti (Figures 1B–1D). It was observed that the deposition of Co occurred in the form of a film, which contrasts with grainy islands reported in the case of Zn, Ag, and Cu following the sputtering protocol.^25^^,^^35^^,^^36^ The elemental analysis using EDS showed the presence of Co on all the deposited surfaces (Table S1). The atomic weight percent of Co increased with deposition time, indicating successful deposition. Figures 1E–1H represents the surface topography of the Ti and Co-deposited Ti discs observed using AFM. The Co-deposited surface showed a uniform thin film formed by coalesced Co nanograins. The 3D AFM images of the Ti and Co-deposited Ti disc are provided in the SI (Figure S1). Figure 1I shows a photograph of control and Co-deposited discs. From the images, the color of the Ti samples changed from light to dark brown with increased sputtering time, attributed to the optical properties of the Co film. The surface roughness of Ti reduced after Co deposition, and the average surface roughness of Ti, Ti-Co_5_, Ti-Co_10_, and Ti-Co_15_ was found to be 12, 4.8, 5.3, and 2.4 nm, respectively (Figure 1J). The reduction in surface roughness after Co deposition could be due to the filling of the surface irregularities by the Co nanograins. The surface profilometry data revealed the formation of A uniform 246 nm Co oxide thin film within 15 min of DC sputtering.

### Total metal content and ion release

**Figure 2:**
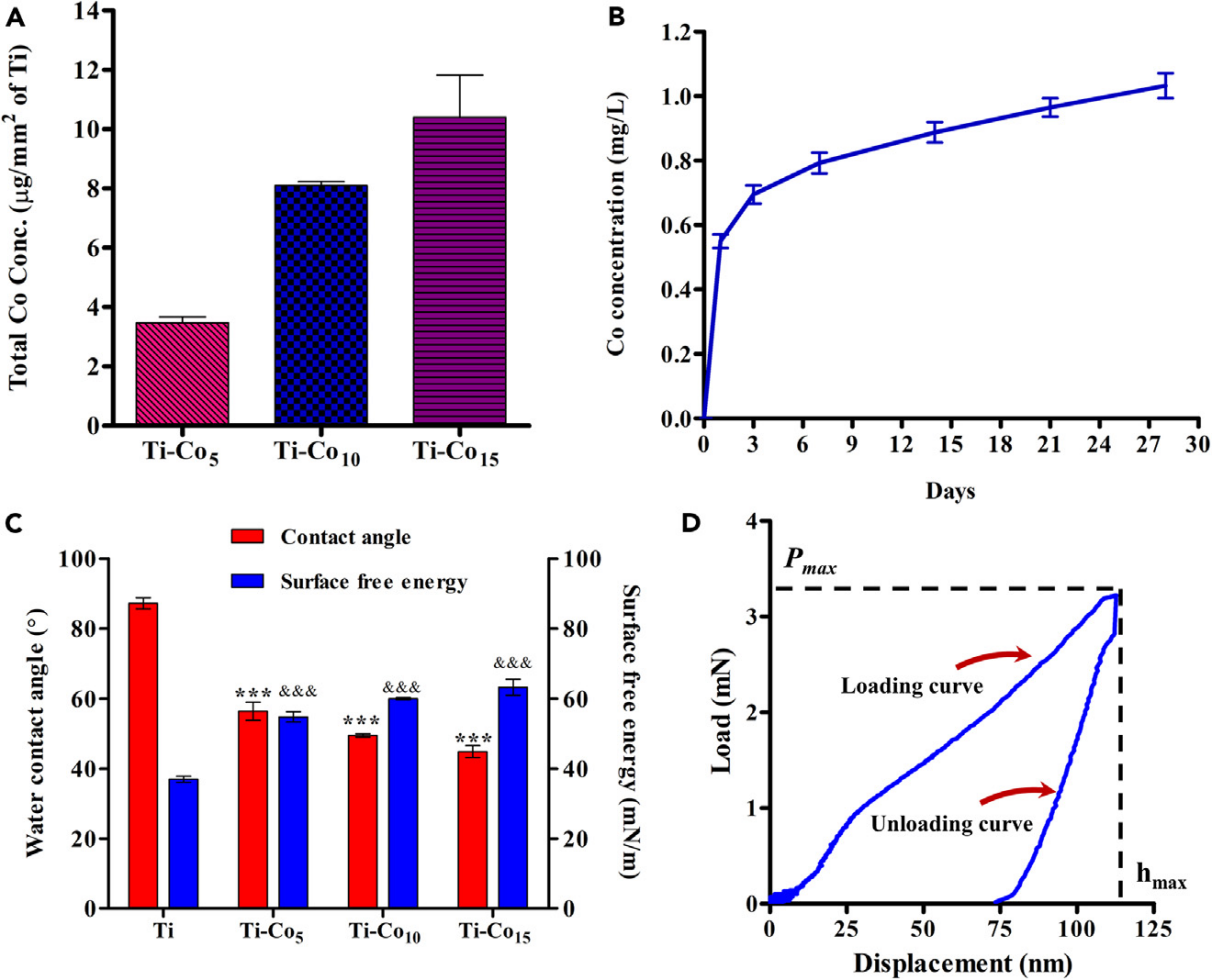
Physicochemical characteristics of Co deposited Ti discs Quantitative estimation of total Co content deposited on Ti discs (A). Evaluation of Co ion release from Ti-Co_15_ surface over 28 days (B). Assessment of water contact angle and surface free energy of the Ti and Co-deposited Ti discs (C).Force-displacement curve of Ti-Co_15_ surface using nanoindentation technique (D). ∗∗∗ and &&& indicate p < 0.001 when compared with Ti.

The total Co content on the different sample surfaces was analyzed using AAS. After 5, 10, and 15 min of deposition, the Co content of the Ti surfaces was 3.47, 8.10, and 10.41 μg/mm^2^, respectively (Figure 2A). From the graph, it is evident that a time-dependent increase in the Co content was observed. These results are in good agreement with the results of the EDS analysis (Table S1). Figure 2B illustrates the release of Co from the Ti-Co_15_ sample over 28 days. A biphasic ion release profile was observed in which an initial burst of Co around 24 h was followed by a sustained release up to 28 days. It is worth noting that the cumulative release was only 12.6%. The lower release of Co from the Ti-Co_15_ sample indicates strong binding between the Co coating and the Ti surface. Such long-term release of Co in the peri-implant microenvironment could be a possible approach to preventing infection after surgery and the subsequent implant healing period. However, this would warrant *in vivo* studies.

### Surface wettability

Physical adsorption of plasma proteins from the blood to the implant surface is one of the earliest biological events that govern osteoblast attachment and osseointegration success.^37^ Wettability and SFE of the implant surface have been shown to play a significant role in initial protein adsorption.^38^ As shown in Figure 2C (red bars), the control Ti disc exhibited a WCA of 87°, whereas Ti-Co_5_, Ti-Co_10_, and Ti-Co_15_ showed WCAs of 56.4, 49.5, and 44.8°, respectively. In the case of Ti-Co_5,_ the WCA is reduced by ∼30°, which is further reduced with time. The decrease in WCA can be attributed to the physicochemical properties of Co-deposited Ti discs, such as topography, SFE, and chemical composition. As per the Owens-Wendt geometric mean equation, the calculated SFE of Ti, Ti-Co_5_, Ti-Co_10_, and Ti-Co_15_ were 37, 54.8, 60, and 63 mN/m, respectively (Figure 2C, blue bars). A study by Hao et al. (2014) showed that the intermediate wettability of 20–70° promoted the attachment, spreading, and osteogenic differentiation of hMCSCs.^39^ Authors found that this moderate wettability promoted the expression of αvβ_1_ integrins in the MSCs, which could be the probable reason for improved osteogenesis. In our study, the better cell attachment, spreading, and expression of osteogenic genes on Co-deposited Ti discs could be due to 1) nanoscale surface topography, 2) improved surface wettability and SFE, and 3) the chemical properties of the surface. Overall, Co deposition on the Ti discs reduced the WCA and increased SFE compared to the control, which further proves the suitability of Co coating on dental implant materials.

### Nanomechanical properties

Nanoindentation is a technique to assess the nanomechanical properties of thin coatings on biomaterials.^40^ It was used to evaluate hydroxyapatite, TiN, TiB, and Cu-Ag coatings on the Ti surface.^40^^,^^41^^,^^42^^,^^43^ Figure 2D displays the load-displacement curves obtained from loading and unloading the Berkovich indenter onto the Ti-Co_15_ surface. The Ti-Co_15_ surface revealed an average hardness of 39.10 GPa and an average elastic modulus of 438.7 GPa (Table S2). The surface coatings applied to the biomaterials should possess high mechanical properties to ensure their stability during clinical use.^43^ The results showed the formation of a hard Co thin film on the Ti surface, which would resist mechanical damage during implantation.

### Surface chemical composition

**Figure 3:**
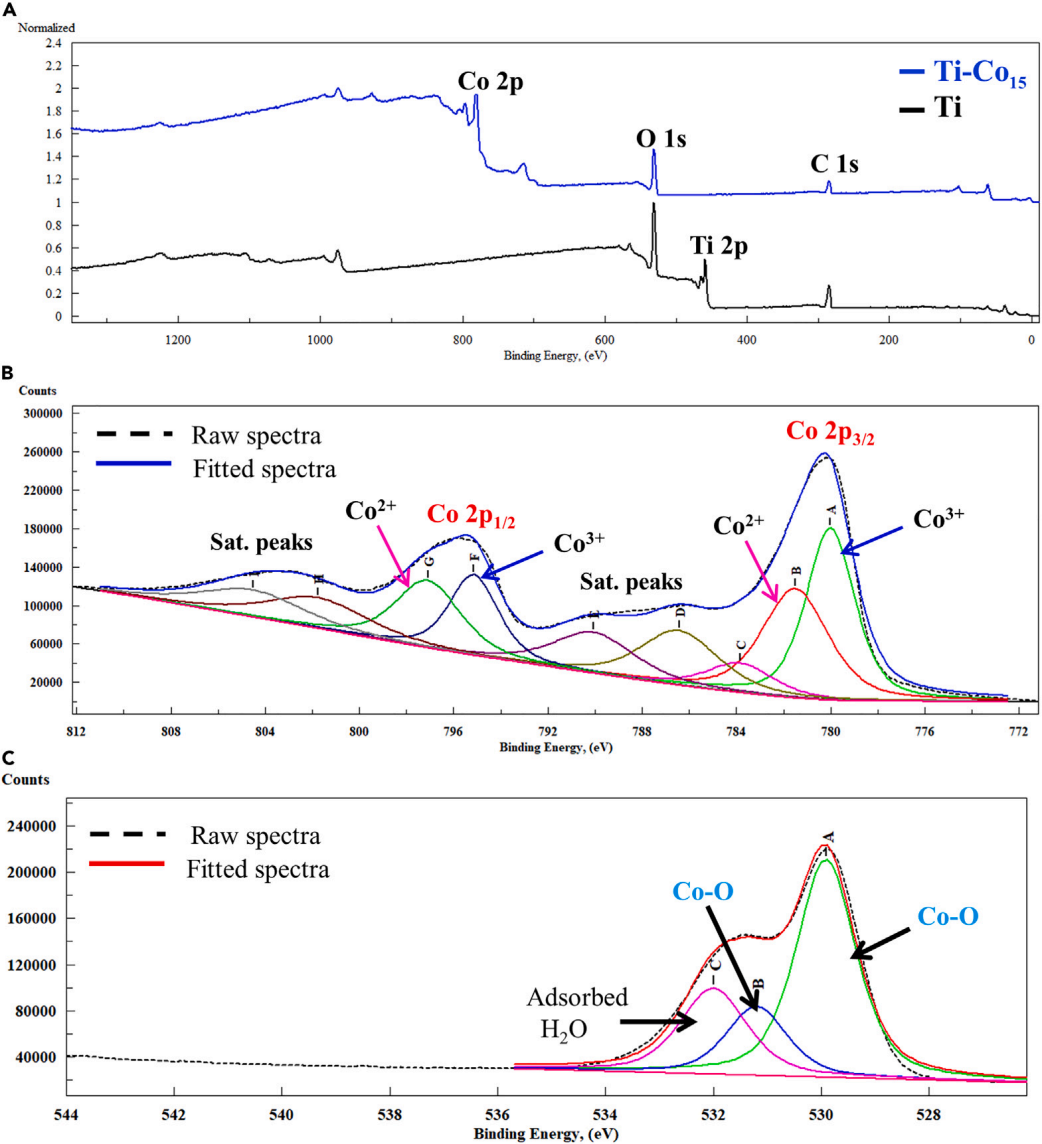
XPS survey spectra of Co deposited Ti discs XPS survey spectra of Ti (black line) and Ti-Co_15_ (blue line) (A). High-resolution deconvoluted Co 2p spectra (B). High-resolution deconvoluted O 1s spectra (C).

XPS was employed to assess the surface chemical composition of the Ti and Co-deposited sample (Ti-Co_15_). The XPS survey spectra of Ti showed prominent peaks at 285, 531, and 459 eV, which could be assigned to C 1s, O 1s, and Ti 2p, respectively, as shown in Figure 3A. The peak at 284.8 eV on the Ti surface could be due to carbon contamination. The Ti-Co_15_ also showed C 1s and O 1s peaks at 285 and 531eV. The peaks at 781 and 796 eV were also seen and could be assigned to characteristic Co 2p_3/2_ and Co 2p_1/2_ doublet, confirming the Co deposition on the Ti disc. Due to the Co deposition, the characteristic Ti 2p peak at 458 eV was not detected in the Ti-Co_15_ survey spectra. Figure 3B indicates the Co2p core-level spectra. The first pair of Co 2p_3/2_-2p_1/2_ peaks at 780 and 795.1 eV could be assigned to the Co^2+^ oxidation state. Similarly, the second pair of Co 2p_3/2_-2p_1/2_ peaks at 781.5 and 797.1eV could be attributed to the Co^3+^ oxidation state. Also, the corresponding O 1s spectrum showed peaks at 529.6 and 531.4 eV, corresponding to the Co-O bond (Figure 3C).^44^ The additional peak at 532 eV could be attributed to water adsorbed on the Ti-Co_15_ surface (Figure 3C). The XPS data is in agreement with the previous research on Co_3_O_4_. Thus, the thin film contains Co_3_O_4_ as a major chemical species. The detection of Co_3_O_4_ in the coating might be due to the Co ions interacting with traces of oxygen in the chamber and further exposure to air under ambient conditions.

### *In vitro* antibacterial assay

**Figure 4:**
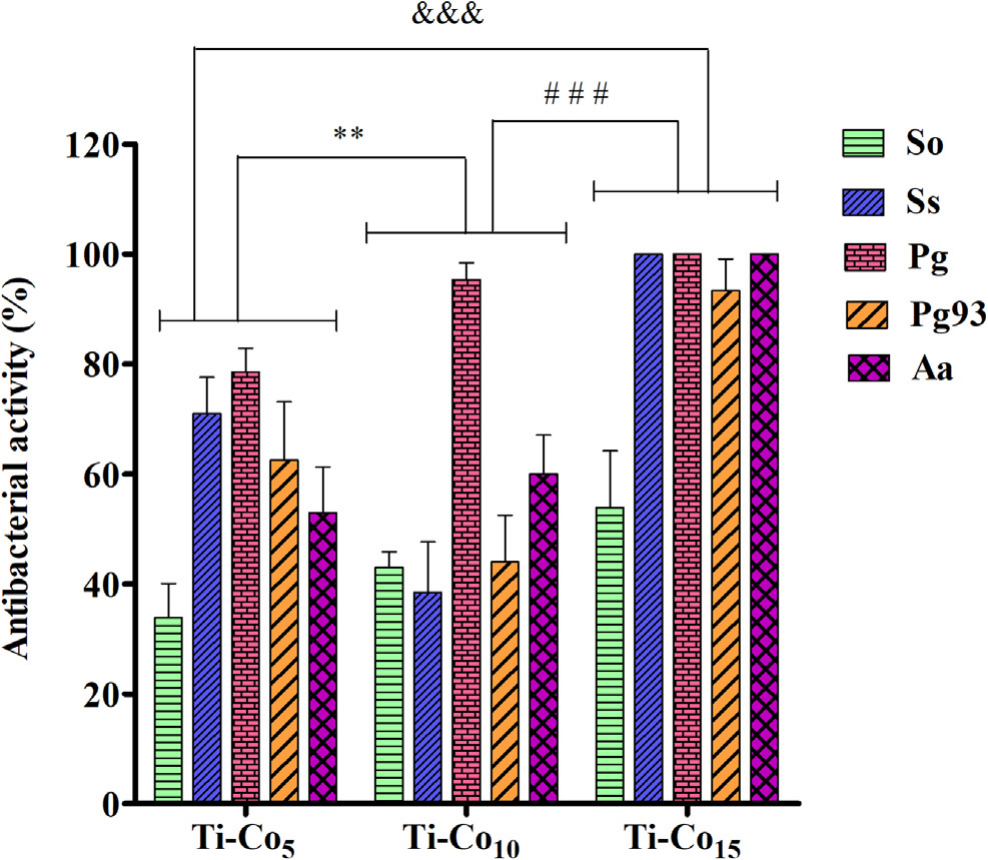
Antibacterial activity of Co-deposited Ti discs against periodontal pathogens ∗∗ indicates p < 0.01, when compared to Ti-Co_5_, ### indicates p < 0.001 when compared to Ti-Co_10_, and &&& indicates p < 0.001 when compared to Ti-Co_5_.

The antimicrobial activity of Co-deposited Ti discs was evaluated against the periodontal pathogens by the modified JIS Z 2801 method. The total viable count (TVC) analysis showed 100% antibacterial activity of Ti-Co_15_ against Ss, Aa, Pg, Pg 93, and 53% against So (Figure 4). The antibacterial effects of Ti-Co_5_ and Ti-Co_10_ were lower (compared to Ti-Co_15_) hinting at a concentration-dependent antibacterial activity. The antibacterial activity possibly correlates with the non-uniform nature of the Co thin films when the sputtering time was 5, 10 min. The antibacterial effect of the Co-deposited surface against each pathogen was statistically significant. In the past, Co-containing metal-organic frameworks have been used as antibacterial compounds and have shown promising bactericidal effects on Gram-positive and Gram-negative bacteria.^45^^,^^46^^,^^47^^,^^48^ A Co-based multifunctional wound dressing has shown potent concentration-dependent antibacterial activity against *Staphylococcus aureus* and *Pseudomonas aeruginosa*.^33^ Mycosynthesized Co oxide nanoparticles using *Aspergillus brasiliensis* showed more or less similar antimicrobial potential against Gram-positive (*Bacillus subtilis* and *Staphylococcus aureus*) and Gram-negative (*Pseudomonas aeruginosa* and *Escherichia coli*) bacteria compared to ampicillin, streptomycin, gentamycin, and erythromycin.^49^ To the best of our knowledge, the results on the broad-spectrum antibacterial activity of Co on bacteria implicated in peri-implant infections are being reported for the first time.

**Figure 5:**
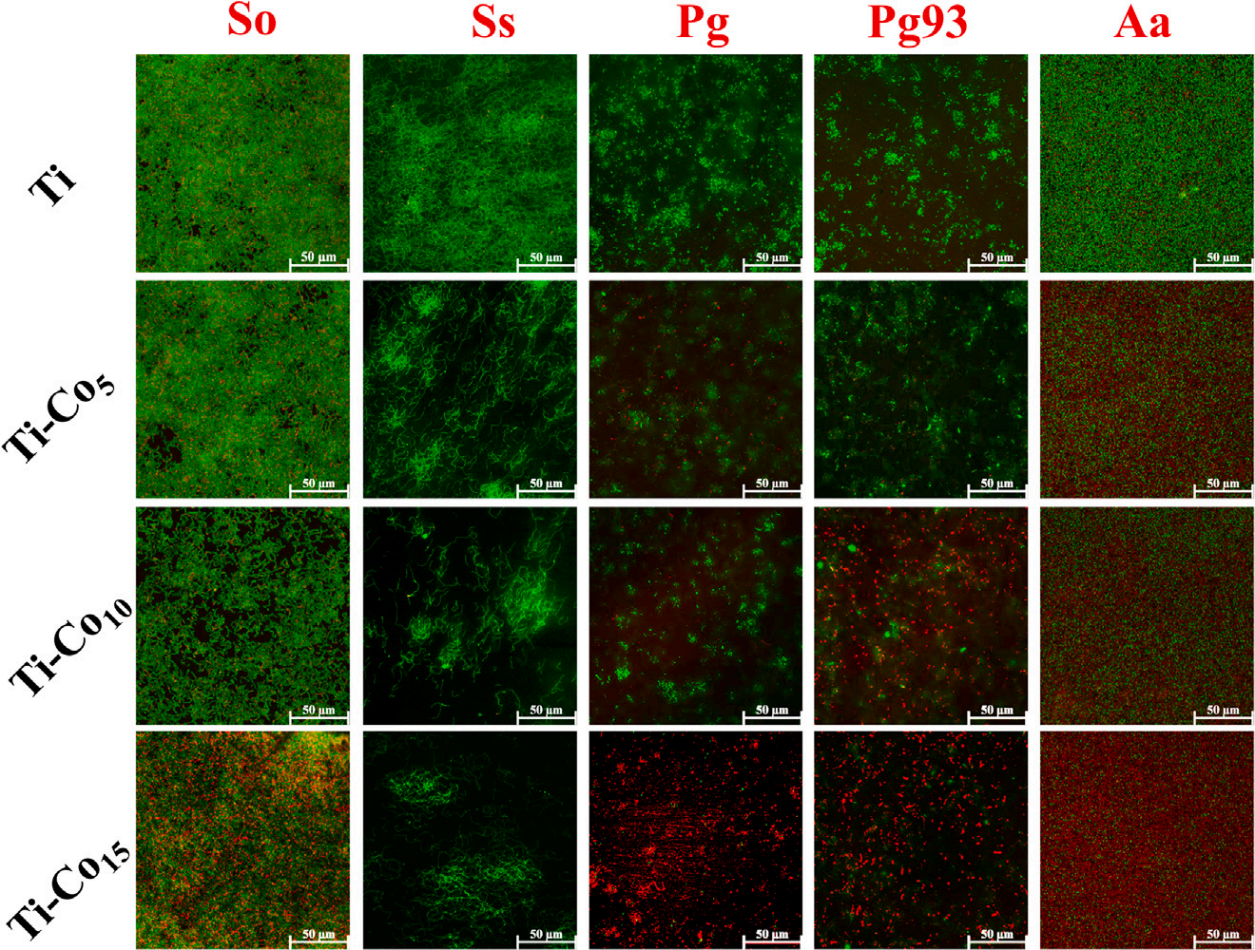
Live-dead staining of periodontal pathogens grown on Ti and Co-deposited Ti discs Scale bar represents 50 μm.

A qualitative evaluation of live and dead cells on Ti and Co-deposited surfaces was performed using confocal microscopy. A reduction in live bacteria (green fluorescence) on the Co-deposited surfaces after 24 h of incubation was observed compared to the control (Figure 5). Ti-Co_15_ surface showed a significant reduction in cell number compared to Ti, Ti-Co_5,_ and Ti-Co_10_. The antiadhesive activity data further supports the TVC assay results, hinting at contact killing of the bacteria due to Co released from the surface.

**Figure 6:**
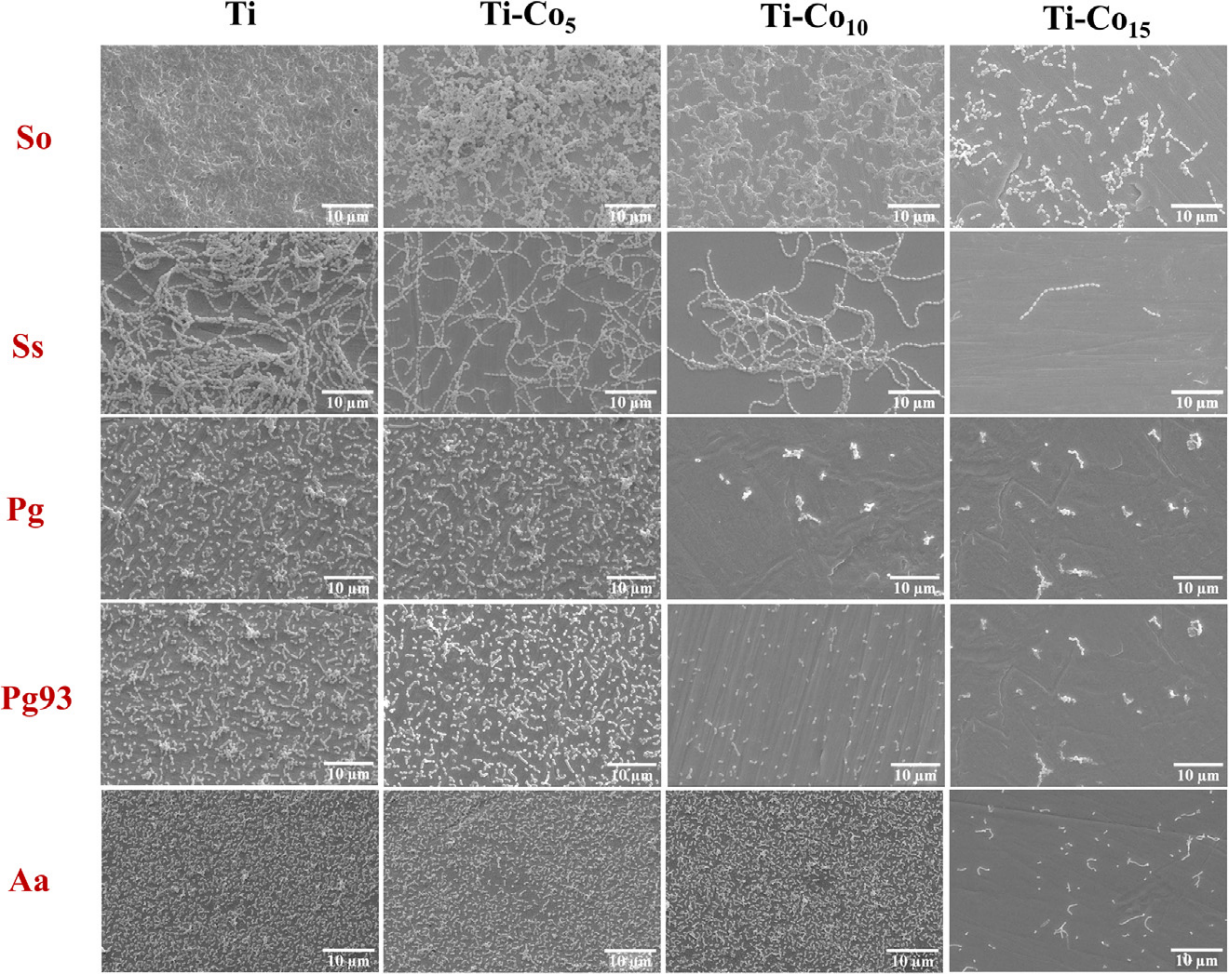
Scanning electron microscopy images of periodontal pathogens on Ti and Co-deposited Ti discs Scale bar represents 10 μm.

Figure 6 shows SEM images of periodontal pathogens grown on Ti and Co-deposited samples. It was observed that the Ti-Co_15_ surface causes a significant reduction in bacterial cell population compared to the control. In addition to this, the Ti-Co_15_ surface clearly showed a change in bacterial cell morphology and cell surface damage. Likewise, Ti-Co_5_ and Ti-Co_10_ samples showed only a slight reduction in bacterial cell number compared to Ti. The substantial bacterial cell reduction on the Ti-Co_15_ surface could be attributed to the release of Co ions at high concentrations, rendering the surface unsuitable for bacterial cell attachment. Overall, the live-dead staining and SEM substantiate the TVC data.

### *In vitro* biocompatibility

#### Cell viability and proliferation assay

**Figure 7:**
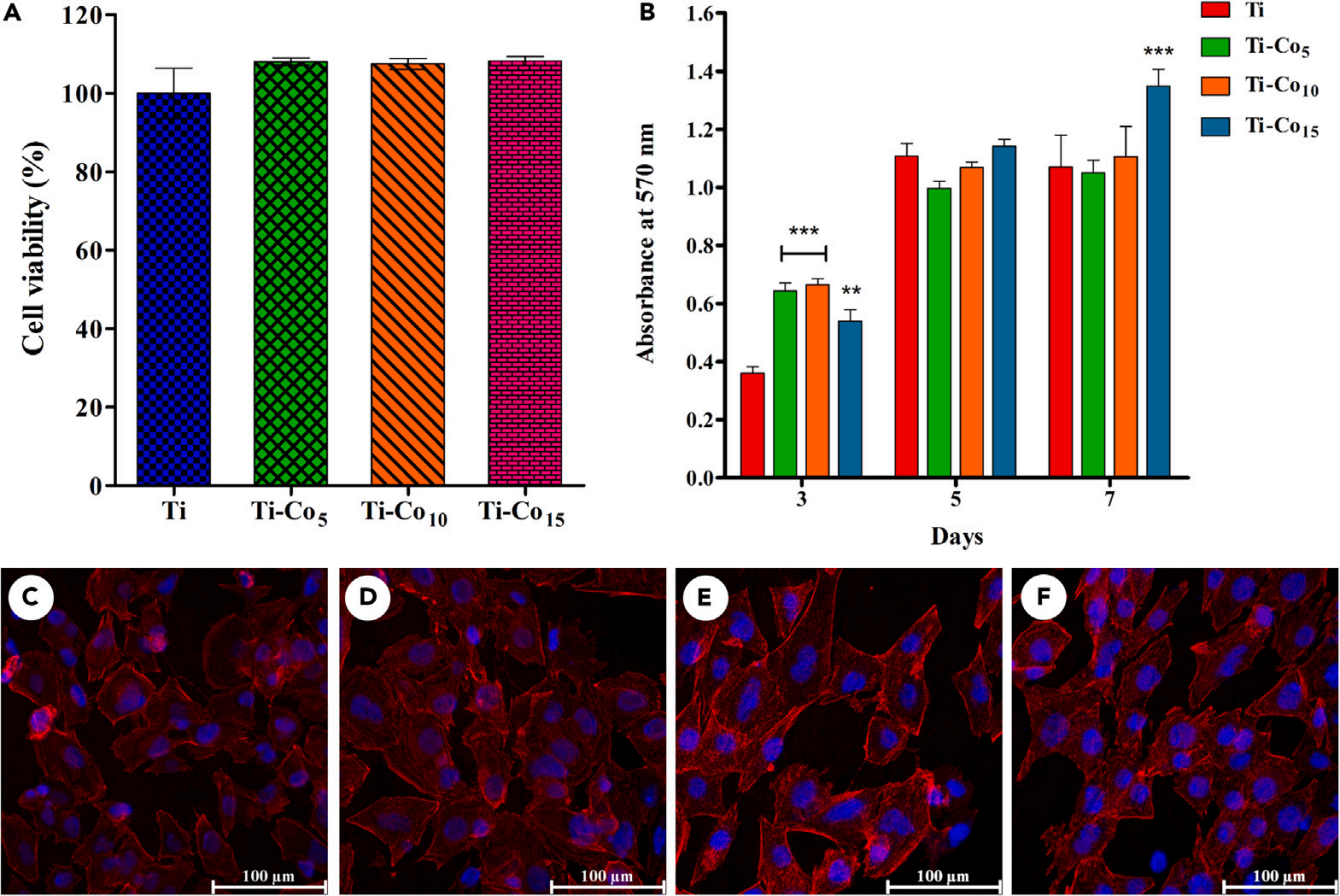
Viability, proliferation and visualization of MG-63 cells cultured on Co-deposited Ti discs Percentage cell viability of MG-63 cells cultured on Ti and Co-deposited Ti discs (A). Cell proliferation ability of MG-63 cells cultured on Ti and Co-deposited Ti discs (B). ∗∗ indicates p < 0.01, and ∗∗∗p < 0.001 when compared with Ti. Confocal microscopic images of the cytoskeleton and nuclear staining of MG-63 cells cultured on Ti (C), Ti-Co_5_ (D), Ti-Co_10_ (E), and Ti-Co_15_ (F) for 24 h. Scale bar represents 100 μm.

Osteoblast cell cultures have been widely used to understand bone-biomaterial interactions. Different osteoblast cell lines, such as MG-63, SaOs-2, MC3T3-E1, and primary human osteoblasts, have been employed to study the effect of implant surface modification on their bioactivities^50^^,^^51^^,^^52^^,^^53^ Figure 7A shows the percentage cell viability of MG-63 cells on Ti and Co-deposited Ti samples after 24 h of culturing. A statistically significant difference in the cell viability was not observed between Ti and Co-deposited Ti samples indicating the excellent cytocompatibility of the Co-deposited surfaces.

Figure 7B indicates the results of the cell proliferation assay. All the Co-deposited Ti surfaces supported the proliferation of MG-63 cells at day 3 compared to the Ti surface. However, the cell proliferation data on day 5 showed a statistically insignificant difference. A higher absorbance, indicating cell proliferation, was observed on day 7 on the Ti-Co_15_ surface compared to the Ti, Ti-Co_5_, and Ti-Co_10_ surfaces. Thus, it is interesting to note that Co deposition on Ti surfaces favored the proliferation of MG-63 cells. The proliferation data supports the applicability of Co for dental implant surface modification.

### Cell attachment and spreading behavior

The attachment and spreading of osteoblasts on the Ti surface is the first key event that profoundly influences cell proliferation and differentiation.^54^ As evidenced by the cell morphology, the spreading behavior of MG-63 cells on the Co-deposited Ti surface was much different from that of those grown on the Ti surface after 24 h. As seen in Figure 7C, the MG-63 cells grown on the control surface appear spindle-shaped, flat polygonal in shape, with a few round cells. Morphologically, the cells grown on Ti-Co_5_ and Ti-Co_10_ surfaces were more or less similar to those on Ti (Figures 7D and 7E). Contrary to this, the cells grown on the Ti-Co_15_ surface appeared large, flat, and polygonal with excellent spreading, almost covering the entire deposited surface (Figure 7F). The differences in cell attachment and spreading behavior on the Ti-Co_15_ surface can be attributed to improved wettability and nanoscale roughness.^55^ Moreover, the enhanced cell attachment and spreading of osteoblast on the Ti-Co_15_ surface could speed up the contact osteogenesis process (i.e., *de novo* bone formation on the implant surface), ensuring the early and long-term survival of the implant.

### Monitoring the alkaline phosphatase activity

**Figure 8:**
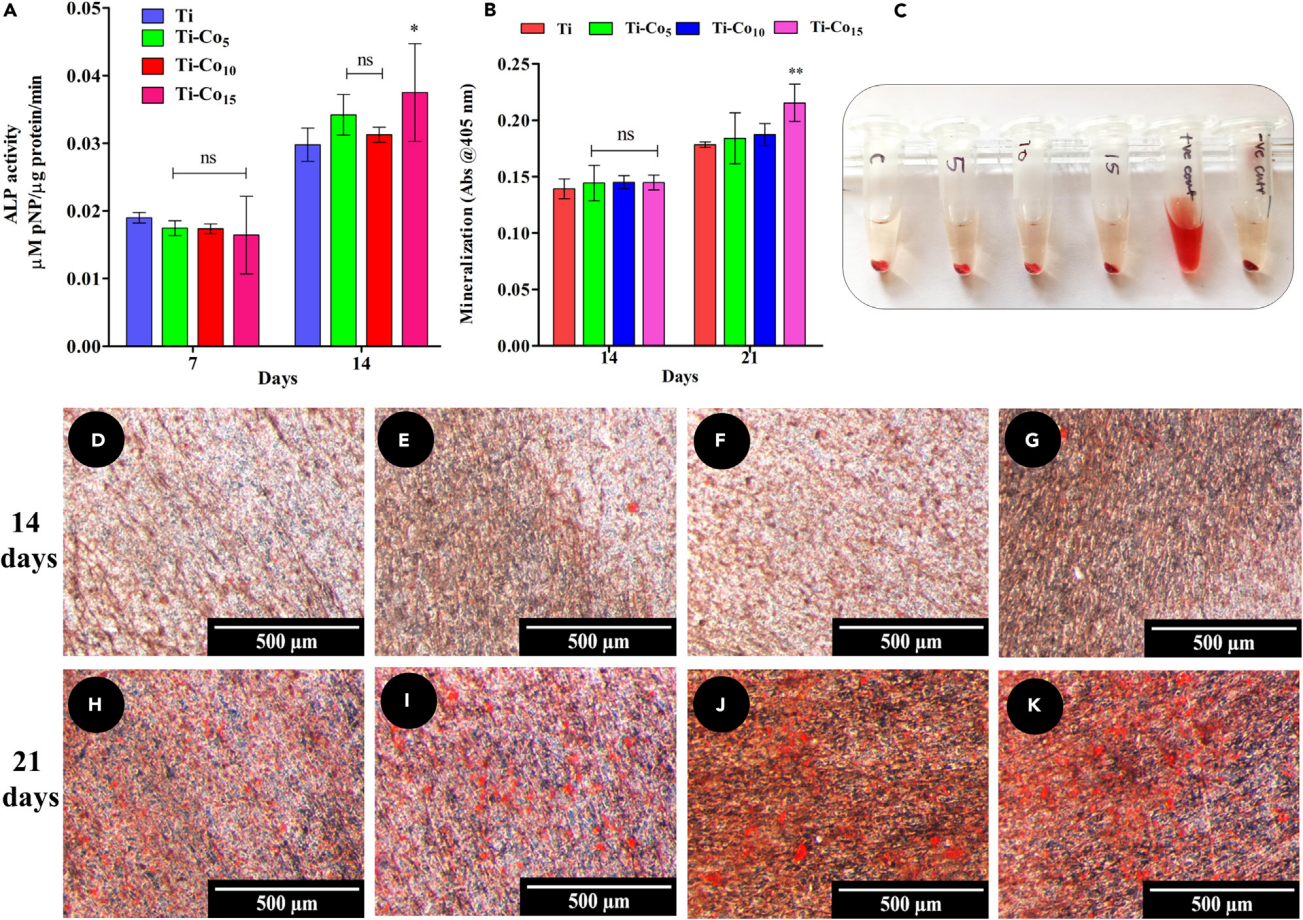
Alkaline phosphatase activity of MG-63, hemocompatibility of Co-deposited Ti and calcium mineralization on Co-deposited Ti Determination of ALP activity (A). Spectrophotometric estimation of ARS dye extracted from cell monolayer (B). ∗ Indicates p < 0.05, ∗∗p < 0.01, when compared with Ti. Photographic image of microcentrifuge tubes containing blood sample kept in contact with Ti, Co-deposited Ti, Triton X-100 (positive control), and saline (negative control) (C). Stereo microscopic images of ARS stained MG-63 cells cultured on Ti (D), Ti-Co_5_ (E), Ti-Co_10_ (F), Ti-Co_15_ (G) for 14 days and Ti (H), Ti-Co_5_ (I), Ti-Co_10_ (J), Ti-Co_15_ (K) for 21 days. Scale bar represents 500 μm.

ALP is an important enzyme involved in bone formation and is a marker of early osteogenic differentiation.^56^ The data on the ALP activity of MG-63 osteoblasts cultured on Ti and Co-deposited Ti surfaces for 7 and 14 days are depicted in Figure 8A. On day 7, the difference in the ALP activity between the control and Co-deposited Ti was not statistically significant. However, on day 14, significantly higher ALP activity (p < 0.05) was observed on Ti-Co_15_ compared to Ti, Ti-Co_5_, and Ti-Co_10_. Recently, Co-doped tricalcium phosphate scaffolds have been shown to promote proliferation with a substantial increase in the ALP activity of human bone marrow stromal cells (BMSCs).^57^ In a similar study, Co incorporated hydroxyapatite (HA) scaffold (Co-HA, 1.25% Co content) supported more ALP activity of MG-63 cells compared to the HA scaffold.^58^ Thus, the results in the present study are in agreement with earlier reports.

### Assessment of calcium deposition

The mineralization efficiency of MG-63 cells on Ti and Co-deposited Ti surfaces was assessed by performing an ARS assay. After 14 days, no significant difference in the mineralization efficiency of the MG-63 cells was observed between Ti and Co-deposited Ti surfaces, as shown in Figures 8D–8G and B. At 21 days, red-colored calcium-rich deposits were observed on all the surfaces (Figures 8H–8K). Interestingly, the distribution of calcium-rich deposits was observed over the entire Ti-Co_15_ surface compared to others (Figures 8K and 8B). As shown in Figure 8B, quantitative measurements (ARS) substantiated the enhanced calcium deposition on the Ti-Co_15_ surface compared to Ti, Ti-Co_5_, and Ti-Co_10_ surfaces. The increase in extracellular matrix (ECM) mineralization observed in the Co-deposited Ti indicates improved mineralization potential of the discs. The data indicate good bioactivity of osteoblasts post-attachment on the Co-deposited surface.

### Monitoring the expression of osteogenesis-related genes

**Figure 9:**
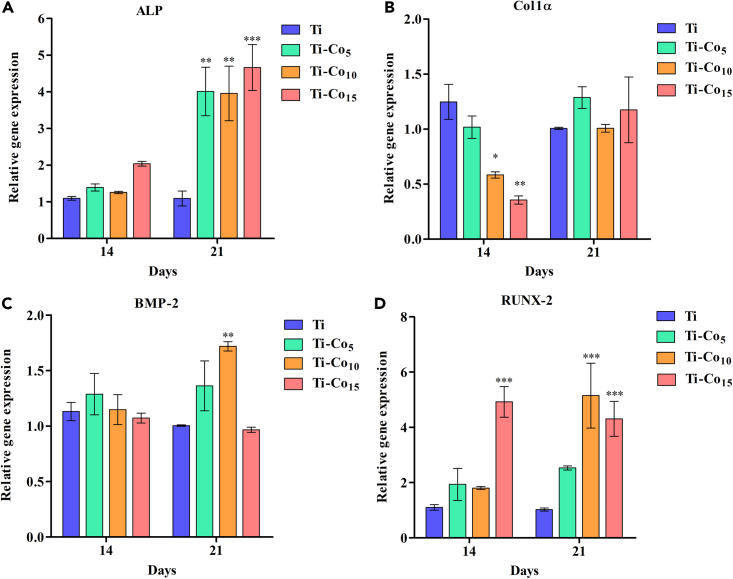
Temporal expression of osteogenesis-related genes in MG-63 osteoblasts cultured on Ti and Co-deposited Ti discs Relative gene expression analysis of osteogenesis-related genes: *ALP* (A), *Col 1α* (B), *BMP-2* (C), and *RUNX-2* (D) in MG-63 osteoblasts cultured on Ti and Co-deposited Ti samples for 14 and 21 days ∗ indicates p < 0.05, ∗∗p < 0.01, ∗∗∗p < 0.001 when compared with Ti.

Co is an essential trace element that the human body requires and is an integral part of vitamin B12.^59^ Metals such as Mg, Zn, and Sr have been used for the surface modification of dental implants to improve their osteogenic potential.^60^^,^^61^^,^^62^ Earlier studies reported that Co induces a hypoxia-like response and activates the hypoxia-inducible factor 1-alpha, which further controls the expression of vascular endothelial growth factor, which is a potent angiogenic factor.^63^^,^^64^^,^^65^ The effect of Co deposition on the expression of *ALP*, *Col1α*, *BMP-2*, and *RUNX-2* (osteogenesis-related) genes in MG-63 osteoblasts was assessed by qPCR (Figure 9). ALP is responsible for the formation of hard tissue; it hydrolyzes pyrophosphate and provides inorganic phosphate to facilitate ECM mineralization. BMP-2 recruits undifferentiated mesenchymal stem cells and differentiation into osteoblast in early osteogenesis. Col1α is an inherent part of the extracellular bone matrix. RUNX-2 is an important transcription factor that controls downstream osteogenic gene expression.^66^ We did not observe a statistically significant difference in the expression of the *ALP* gene between Ti and Co-deposited Ti surfaces after 14 days. However, on day 21, a significantly higher *ALP* gene expression was observed on all Co-deposited Ti surfaces compared to control (p < 0.01 for Ti-Co_5_ and Ti-Co_10_, p < 0.001 for Ti-Co_15_) (Figure 9A). On day 14, for Ti-Co_10_ and Ti-Co_15_ surfaces, the relative gene expression of *Col1α* was significantly low (Ti-Co_10_ p < 0.05, and Ti-Co_15_ p < 0.01). Whereas the expression of *Col1α* was restored on day 21 with no statistically significant differences between native Ti and Co-deposited surfaces (Figure 9B). Also, the relative gene expression of *BMP-2* was similar on the Co-deposited and non-deposited surfaces after 14 and 21 days. However, on day 21, the Ti-Co_10_ surface showed significantly higher *BMP-2* expression (p < 0.01) than Ti (Figure 9C). In the case of *RUNX-2*, a significantly higher gene expression was observed on the Ti-Co_15_ sample (p < 0.001) at the 14^th^ and 21^st^ day compared to the Ti (Figure 9D).

### Hemolysis assay

Blood compatibility is one of the essential criteria that biomaterials must satisfy before clinical application. Blood incompatibility of biomaterial may lead to severe complications such as hemolysis, coagulation, and immune rejection, ultimately resulting in implant failure.^67^ The hemocompatibility of Co-deposited Ti was studied using a hemolysis assay. Figure 8C displays the results of the incubation of blood with Ti and Co-deposited Ti discs for 1 h. Ti and all Co-deposited surfaces revealed <5% hemolysis compared to Triton X-100 (positive control, complete hemolysis) (Figure S2). These data suggested that Co-deposited surfaces possess excellent hemocompatibility.

## Discussion

Ti is widely used for the fabrication of dental implants, but it lacks effective bioactivity and antibacterial activity, which delays the osseointegration process. Ti alloys possess a higher elastic modulus compared to natural the bone which is responsible for the stress shielding effect, but these were shown to release cytotoxic aluminum and vanadium ions.^68^ These issues were resolved by the design of β-type Ti alloys, containing higher amount of β phase stabilizers such as Mo, Zr, and Ta. Ti-15Mo, Ti-24Nb-4Zr-8Sn, and Ti-35Nb-2Ta-3Zr, are some examples of the latter.^69^ The porous β-type Ti alloys can be prepared using powder metallurgy, additive manufacturing (3D printing), and FAST-forge.^69^ Though these alloys have excellent physico-chemical properties they lack effective bioactivity. Thus, continued research to improve the antibacterial properties and osseointegration potential of the Ti implant will essentially promote the use of implants as a long-lasting treatment for edentulous individuals. In the past various techniques were used to modify the Ti surface and coating with Ag, Cu, Zn, Sr, Ga, Sn, and Au conferred antibacterial activity.^70^ Amongst physical techniques, sputtering, has been widely used for the deposition of metals on different surfaces owing to ease of use, achieving the purity of coating, and uniform film deposition.^22^ Few investigations have reported the broad-spectrum antibacterial activity of Co.^45^^,^^46^^,^^47^^,^^48^ Recently, Co-Cr alloy was shown to maintain the tribological properties and corrosion behavior in acidic artificial saliva-lubricated conditions, which implies the possible use of such alloy in dental implant construction.^71^ In the time-course experiment, an increase in Co content with deposition time was observed, suggesting the success of sputtering in the surface modification. It has been shown that osteoblast cells respond to the topographic features of the extracellular matrix such as pits, pores, and fibers with dimensions ranging between 5 and 200 nm.^72^ In addition, the AFM images of Co-deposited Ti surfaces, (especially Ti-Co_15_), show distinct Co nanograins (30–70 nm diameter) which might be a parameter contributing to improved cell attachment. Also, it has been reported that nanoscale surface topography promotes protein adsorption and conformation which in turn influences cell attachment.^73^ Additionally, Co deposition improved the hydrophilicity and SFE of the native Ti surface. Taken together, earlier findings corroborate the results of the present study on Co-deposited Ti using DC sputtering as a dental implant material.

Peri-implantitis is the most common cause of implant failures, with bacterial infection being the root cause. Gram-negative bacteria from the red complex of periodontal pathogens (*Porphyromonas gingivalis, Treponema denticola, and Tannerella forsythia*) are responsible for severe peri-implantitis.^10^^,^^11^^,^^12^ In this study, we assessed the antibacterial potential of Co-deposited Ti specifically against periodontal pathogens belonging to yellow (*S. sanguinis, S. oralis),* purple (*A. actinomycetemcomitans)*, and red (*P. gingivalis, P. gingivalis 93* (Indian strain)) complexes. It was noteworthy that Co-deposited Ti, especially Ti-Co_15_, showed excellent antibacterial activity against periodontal pathogens. Although metals possess remarkable antibacterial activity with meagre chances of developing resistance in bacteria, the minute concentrations may be cytotoxic to the host cells. Keeping this in mind, we investigated the effect of Co coating on the viability, attachment, spreading, and proliferation potential of the MG-63 osteoblasts. The results showed that Co coating on Ti did not have any cytotoxic effect on MG-63 osteoblasts; the Ti-Co_15_ surface supported more cell attachment and spreading, one of the most critical steps during implant healing.^54^ In addition, the Ti-Co_15_ surface exhibited more ALP activity and mineralization efficiency, reaffirming the possibility of Co coating for the surface modification of Ti to enhance its osseointegration. The qPCR assay revealed that, at 14 days an overall increase in ALP gene expression on the Co-deposited Ti (especially on Ti-Co_15_) compared to control Ti continued up to 21 days. A study by Kargozar et al. showed a similar trend of increase in ALP gene expression when SaOS-2 cells (human osteosarcoma cell line) were treated with Co-substituted bioglass.^74^ Based on these results, it may be concluded that Co ions have a positive effect on the ALP gene expression. In the case of Col1α gene expression, previous studies have shown that higher concentrations of Co ions (0.25–1 mM) had a negative impact on the expression of the Col1α gene in MG-63 cells.^75^^,^^76^ In our investigation we found a reduction in the Col1α gene expression on the Co-deposited Ti at 14 days in a dose-dependent manner i.e., more reduction on Ti-Co_15_ followed by Ti-Co_10_, and Ti-Co_5_ which could be attributed to higher Co release during the initial period of incubation. However, it is important to note that the Col1α gene expression normalized at 21 days probably due to the depletion of Co ions from the surface during incubation. Moreover, MG-63 cells cultured on the Ti-Co_15_ surface showed higher expression of *BMP-2* and *RUNX-2* genes, thus proving the safety of Co coating for *in vivo* use. Surface modification of dental implants using metal nanoparticles provides the advantages of antibacterial activity and improved osseointegration. However, the difficulties in the degradation and excretion of these metal nanoparticles might pose serious health disadvantages such as cytotoxicity, bioaccumulation, and systemic toxicity.^77^ Our findings suggest that although Co-deposited Ti surfaces do not have any cytotoxic effect on the MG-63 osteoblasts, the *in vivo* compatibility studies are warranted to evaluate the excretion, bioaccumulation, and systemic health consequences of Co coating. It is worth noting that the sputter deposition of Co on biomaterial surfaces may prevent biomaterial-associated infections, ensuring successful bio-integration.

### Limitations of the study

In this study, we have demonstrated that the Co-deposited Ti samples possess excellent cytocompatibility against MG-63 osteoblasts. However, we did not study the effect of Co coatings *in vivo*. The *in vivo* study would be an advancement, leading to implants with superior properties.

## STAR★Methods

### Key resources table

**Table:** 

REAGENT or RESOURCE	SOURCE	IDENTIFIER
**Bacterial and virus strains**		

*Streptococcus sanguinis*	https://www.Microbiologics.com	Cat# 0858P
*Streptococcus oralis*	https://www.Microbiologics.com	Cat# 0423P
*Aggregatibacter actinomycetemcomitans*	https://www.Microbiologics.com	Cat# 01175P
*Porphyromonas gingivalis*	https://www.Microbiologics.com	Cat# 0912P
*Porphyromonas gingivalis* 93	Samleene Bio Engineering Pvt. Ltd. India	NA

**Chemicals, peptides, and recombinant proteins**		

SYTO9	Invitrogen	Cat# S34854
Propidium iodide	Sigma Aldrich	Cat# P4864
Glutaraldehyde solution, Grade 1	Sigma Aldrich	Cat# G7651
Dimethylsulfoxide	Spectrochem	Cat# 0104264
Paraformaldehyde	Sigma Aldrich	Cat# 158127
Triton X-100	Sigma Aldrich	Cat# 93443
Rhodamine-phalloidin	Invitrogen	Cat# R415
Hoechst	Invitrogen	Cat# H3570
Alizarin red S dye	Sigma Aldrich	Cat# A5533
p-nitrophenol phosphate	HiMedia	Cat# RM1134
L-Ascorbic acid	HiMedia	Cat# TC094
β-glycerophosphate	HiMedia	Cat# TC463
Dexamethasone	HiMedia	Cat# TC277
TRIzol reagent	Invitrogen	Cat# 15596026

**Critical commercial assays**		

ReadyScript® cDNA synthesis kit	Sigma Aldrich	Cat# RDRT
Pierce™ BCA protein assay kit	Thermo	Cat# 23227

**Experimental models: Cell lines**		

MG-63 Osteoblast	National Center for Cell Science, Pune, India	NA

**Software and algorithms**		

GraphPad prism (version 5.0)	https://www.graphpad.com	NA
Gwyddion (version 2.59)	https://gwyddion.net	NA
Spectral data processor (version 8.0)	https://www.xpsdata.com	NA

**Other**		

DC Sputtering machine (6’’ SPT model)	Hind High Vacuum Company Pvt. Ltd, India	NA
Cobalt sputtering target	Indo-French High-Tech Equipments	NA

### Resource availability

#### Lead contact

Further information and requests for resources and reagents should be directed to and will be fulfilled by the lead contact, Dr. Jyutika M. Rajwade, jrajwade@aripune.org.

#### Materials availability

This study did not generate new unique reagents.

#### Data and code availability

• All data reported in this paper can be made available by lead contact upon request.
• This paper does not report original code.
• Any additional information required to reanalyze the data reported in this paper is available from the lead contact upon request.

### Method details

#### Fabrication of Co-deposited Ti

Circular polished discs of Grade 5 titanium alloy (Ti_6_Al_4_V), 10 mm diameter and 2 mm thickness (Bhagyashali metal, Mumbai, India), were used in the study. The discs were cleaned to remove the surface contaminants from the cutting and machining. Firstly, the discs were washed with a warm detergent solution, followed by five washes with water, and dried. The discs were then treated with acetone, isopropanol, and ethanol for 15 min each in the ultrasonic water bath with three distilled water washes in between and dried. The Co thin film was deposited on the Ti discs using a DC sputtering machine (Hind HiVac, Bangalore, India). Co disc (99.99% pure, diameter 3", thickness 2 mm) was used as a target with a fixed target to substrate distance of 3 cm. The deposition was carried out in an argon (UHP grade) plasma atmosphere (0.1 mbar working pressure and 100 mA current) for varying time intervals (5, 10, and 15 min). Co-deposited samples were abbreviated as Ti-Co_5_, Ti-Co_10_, and Ti-Co_15,_ respectively. The undeposited Ti discs were used as control.

#### Surface morphology and topology analysis

The surface morphology and elemental composition of the samples were determined using scanning electron microscopy (SEM) (MA15 EVO®; Carl Zeiss, Baden-Wurttemberg, Germany) coupled with energy dispersive X-ray spectroscopy (EDS) (Oxford Instruments, Abingdon, United Kingdom). Before analysis, the samples were sputter deposited with a thin gold film, and images were captured at 20kV accelerating voltage.

The samples' surface topography and average surface roughness were measured using atomic force microscopy (AFM) (Innova®, Bruker, Massachusetts, USA). Antimony doped silicon tip (resonance frequency 300 kHz and nominal elastic constant 40 N/m) was used for scanning the surface in tapping mode. Gwyddion (version 2.59), a freeware, was used to visualize the AFM images and determine the average roughness (Ra).

The sputter deposition rate of Co and coating thickness of the Co-deposited Ti was assessed using the surface profilometry technique. The experimental details and results obtained are presented in the supplemental information (SI) section (Figures S3 and S4).

#### Total metal content and release study

Quantitative estimation of the total Co content of the deposited samples was performed using atomic absorption spectrophotometer (AAS) (ContrAA 800D, Analytikjena, Jena, Germany). Initially, all the samples were immersed in 1 mL of concentrated nitric acid (HNO_3_) for 12 h to dissolve the Co coating completely. The resulting solution was diluted up to 10 mL using double distilled water in a standard flask and used directly for estimation.^22^

The release of Co from the Ti-Co_15_ sample was monitored in 10 mM phosphate-buffered saline (PBS, pH 7.2) at different time intervals using inductively coupled plasma-atomic emission spectrophotometry (ICP-AES) (ARCOS, SPECTRO analytical instruments GmbH, Germany) available at Indian Institute of Technology, Mumbai, India. The sample was immersed in 1 mL of PBS and incubated at 37°C. At pre-determined time intervals (0, 1, 3, 7, 14, 21, and 28 days), the solution was withdrawn and replaced with the same quantity of fresh PBS. The resulting solution was digested in concentrated HNO_3_ for 12 h and further diluted to 5 mL in a standard flask using double distilled water and used for the estimation.^22^

#### Wettability study

The wettability and surface free energy (SFE) of the Ti and Co-deposited samples were assessed using contact angle (CA) measurement. The CA measurements were carried out on a drop shape analyzer (DSA25S, Kruss Scientific, Hamburg, Germany) instrument in static mode using double distilled water and di-iodomethane as a wetting medium (2 μL) at room temperature (RT). The representative contact angle is the mean of three independent measurements. The SFE was calculated using Owens-Wendt geometric mean equation.^78^

$$\gamma_{L}\left(1+\cos\;\theta \right)=2\sqrt{\gamma_{S}^{D}\gamma_{L}^{D}}+2\sqrt{\gamma_{S}^{P}}\gamma_{L}^{P}$$

Where θ is the measured contact angle, $\gamma_{L}$ is the surface tension of the liquid, $\gamma_{L}^{D}$ and $\gamma_{L}^{P}$ are dispersive and polar components of the liquids. The SFE $\left[\gamma_{S}=\gamma_{S}^{D}+\gamma_{S}^{P}\right]$ was determined by measuring the CA of water and di-iodomethane.

#### Nanomechanical properties

The nanomechanical properties (hardness and Young's modulus) of the Ti-Co_15_ surface were evaluated by nanoindentation test using a G200 Nano indenter (Agilent, California, United States). A diamond Berkovich indenter was employed based on a continuous stiffness-measuring technology. A maximum load of 5 mN was applied on the surfaces during the test. A total of 10 indentations in randomly selected areas were made, and corresponding force-displacement curves were recorded.^43^ The Young's modulus (*E)* and hardness (*H)* of the samples were then computed using the relationship described by Oliver and Pharr.

#### Surface chemical composition

The surface chemical composition of the Ti and Ti-Co_15_ samples was analyzed using X-ray photoelectron spectroscopy (XPS) (ESCALAB Xi+, Thermo Fisher Scientific, UK). A monochromatic Al Kα X-ray source was used to irradiate the samples (Spot size 900μm diameter). The survey spectra (0-1350 eV) were acquired at pass energy (CAE) of 200 eV and step size of 1.0 eV, whereas a narrow scan of Co 2p was acquired at pass energy (CAE) of 50 eV and step size of 0.1 eV. The ultra-high vacuum in preloc and analysis chambers was maintained at 7x10^-9^ and 5x10^-10^ mbar, respectively. Spectral data processor (SDP v8.0) software was used to analyze survey spectra and deconvolution of the peaks.^22^

#### Antibacterial assays

Antibacterial assays were performed using *Streptococcus sanguinis* ATCC 10556 (Ss)*, Streptococcus oralis* ATCC 6249 (So)*, Porphyromonas gingivalis* ATCC 33277 (Pg)*, Porphyromonas gingivalis93* (Indian strain) (Pg93) and *Aggregatibacter actinomycetemcomitans* ATCC 29522 (Aa) cultures. Ss and So were cultured and maintained in brain heart infusion broth aerobically; thioglycollate broth was used for *Aa*, while Pg strains were grown in ATCC medium 2722 anaerobically. Tryptic soy-serum bacitracin vancomycin (TSBV) agar and Columbia blood agar (supplemented with 5% sheep blood) were used for the plating of Aa and Pg cultures, respectively. All the cultures were grown at 37°C.

The antibacterial activity of Co-deposited Ti discs was assessed using the modified Japanese Industrial Standards (JIS Z 2801:2000) method. This method applies to metals, ceramic, and plastic products. Briefly, the materials were surface sterilized under UV irradiation for 30 min on each side. The bacterial suspension (50 μL) (approx. 10^5^ cells) was inoculated on each sample, covered with a sterile plastic thin film, and incubated in a humidified chamber at 37°C for 24 h. After incubation, the samples were aseptically transferred to 1 mL of sterile PBS, vortexed for 1 min, and sonicated in an ultrasonic water bath for 2 min. Subsequently, 10-fold serial dilutions of this suspension were prepared, and 10μL of each dilution was spot inoculated on respective agar plates and incubated according to the conditions mentioned earlier. Antibacterial activities of the Co-deposited discs were determined by calculating the reduction in colony forming unit per mL (CFU/mL) compared to the control surface. The percent antibacterial activity was calculated using the following formula,

$$\text{Antibacterial}\;\text{activity}\;\left(\%\right)=\frac{\text{CFU}\;\text{of}\;\text{control}\;\text{surface}\;-\;\text{CFU}\;\text{of}\;\text{treatment}\;\text{surface}}{\text{CFU}\;\text{of}\;\text{control}\;\text{surface}}\times 100$$

To evaluate the anti-adhesive activity of Co-deposited Ti, live-dead staining was performed as described by Liu et al. (2016).^79^ For this, 1 mL bacterial suspension (10^5^ cells/mL) was added to each sample and incubated at 37°C for 24 h. After incubation, all the discs were washed with PBS and stained with SYTO 9 (green fluorescence, 480/500) and propidium iodide (PI) (red fluorescence, 560/635) dyes, which label live and dead cells, respectively, for 30 min at RT in dark condition. All the samples were washed with PBS to remove excess stains and observed under confocal laser scanning microscope (CLSM) (TCS SP8, Leica, Wetzlar, Germany). 10% glycerol was used as a mounting medium to avoid drying of samples during imaging.

To substantiate the results, another set was prepared as described above and after incubation, all the discs were washed with PBS, and cells were fixed in 2.5% glutaraldehyde solution for 1 h at RT. Excess glutaraldehyde was aspirated, and the samples were washed twice with PBS. The samples were then sequentially dehydrated in ethanol gradient (50, 70, 80, 90, 95, and 100%, 10 min incubation at each treatment). After dehydration, samples were sputter coated with gold and observed under SEM.^79^

#### Cell viability and proliferation assay

The cell culture study used the MG-63 osteosarcoma cell line (P-30 to P-40). The cells were provided by National Centre For Cell Science (NCCS) Pune, India. The cells were cultured in minimal essential medium (α-MEM) supplemented with 10% Fetal bovine serum and 1% antibiotic and antimycotic solution in 25 cm^2^ tissue culture flask at 37°C, 5% CO_2,_ and 95% relative humidity. At 90% confluency, sub-culturing was done using 0.25% trypsin EDTA solution at 1:3 split ratios. All the chemicals used for cell culture were procured from HiMedia, Mumbai, India.

Cell viability of MG-63 cells on Co-deposited Ti samples was assessed using MTT (3-(4, 5-dimethylthiazol-2-yl)-2-5 diphenyl tetrazolium bromide) (USB, Cleveland, USA) assay.^22^ Initially, 4 x 10^4^ cells were seeded on each sample in 24-well plate. After 24 h, MTT solution (5 mg/mL) was added and incubated for 4 h. The formazan crystals were dissolved in dimethylsulfoxide (DMSO), and the absorbance of the resultant solution was recorded at 570 nm using a multi-well plate reader (Synergy HT, Bio-Tek Instruments Inc., USA). For determination of cell proliferation MG-63 cells were cultured on Ti and Co-deposited Ti samples for 3, 5, and 7 days. After incubation cell viability at each time point was assessed using MTT assay as mentioned above. The percentage cell viability was calculated using the following formula:

$$\text{Cell}\;\text{viability}\;\left(\%\right)=\frac{{\text{OD}}_{570\;\text{nm}}\;\text{Treatment}\;\text{group}}{{\text{OD}}_{570\;\text{nm}}\;\text{Control}\;\text{group}}\times 100$$

#### Cell adhesion and spreading behavior

To visualize cell adhesion and cytoskeletal architecture of the MG-63 cells grown on Ti and Co-deposited Ti surfaces, cell suspension (80 μL) containing 2 x 10^4^ cells was seeded on each sample, kept in a 24-well plate. After incubation at 37°C in a humidified atmosphere of 5% CO_2_ for 2 h (to facilitate initial cell attachment), 1 mL of medium was added, and incubation was continued for 24 h. Before staining, the samples were fixed using 4% paraformaldehyde (PFA) for 15 min and permeabilized in 0.1 % Triton X-100 for 5 min. Cells were stained with rhodamine-phalloidin (actin stain, 30 min) and Hoechst (nuclear stain, 10 min) dyes and observed under CLSM. Samples were washed twice with PBS after each step.^79^

#### Monitoring alkaline phosphatase (ALP) activity

Intracellular ALP activity of MG-63 cells cultured on Ti and Co-deposited Ti samples were assessed spectrophotometrically by determining the enzymatic conversion of p-nitrophenol phosphate (pNPP) to yellow-coloured p-nitrophenol (pNP) as described by Wang et al. (2016).^80^ Initially, 2 x 10^4^ cells were cultured for 7 and 14 days on the Ti, and Co-deposited Ti surfaces. After incubation, samples were washed with PBS, and cells were lysed using RIPA buffer for 1 h at 4 °C. Lysates were centrifuged at 12000 rpm for 30 min at 4 °C, and the supernatant was used for the assay. 50 μL of lysate was added to 100 μL of pNPP solution (10 mM Tris buffer, pH 9, and supplemented with 1 mM MgCl_2_) and incubated at 37 °C for 30 min. The absorbance of the coloured product was recorded at 405 nm, and the amount of p-nitrophenol formed was estimated using a standard curve. The enzyme activity was normalized to total protein content measured using BCA assay.

#### Assessment of calcium deposition

The effect of Co deposition on the mineralization efficiency of MG-63 cells was assessed using alizarin red S (ARS) dye. Cells (2 x 10^4^) were seeded on the Ti and Co-deposited Ti samples and allowed to proliferate in α-MEM until monolayer formation (6 days). The normal growth medium was replaced with osteogenic medium (α-MEM supplemented with 50 μg/mL ascorbic acid, 10 mM β-glycerophosphate, and 100 nM dexamethasone) in which the cells were grown for 14 and 21 days. Before staining, the monolayer was washed with PBS and then fixed with 4% PFA for 15 min at RT, followed by washing with PBS to remove excess PFA. ARS dye (40 mM, pH 4.2, 500 μL) was added to each sample and incubated for 20 min. Subsequently, the unbound dye was removed, and the stained monolayer was washed five times with distilled water and visualized under a stereomicroscope (Leica M205A, Wetzlar, Germany). For quantitation, stained monolayers were stored at -20°C. The dye was extracted in 500 μL of 10% (w/v) cetylpyridinium chloride solution for 1 h, and then absorbance of the coloured solution was recorded at 405 nm.^81^^,^^82^

#### Monitoring the expression of osteogenesis-related genes

The relative expression of osteogenesis-related genes *viz*, Alkaline phosphatase (*ALP*)*,* Collagen 1 (*Col1α*)*,* Bone morphogenetic protein 2 (*BMP-2*), and Runt-related transcription factor 2 (*RUNX-2*) were studied by the real-time qPCR. Glyceraldehyde 3-phosphate dehydrogenase (*GAPDH*) was used as the reference gene. After 14 and 21 days of incubation, the total RNA was extracted using TRIzol reagent (Invitrogen Life Technologies, Carlsbad, CA, USA). Subsequently, the complementary DNA (cDNA) was synthesized from 1 μg of total RNA using ReadyScript® cDNA synthesis kit (Sigma-Aldrich, St. Louis, United States). The qPCR was performed using LightCycler® 480 SYBR Green I Master mix on the LightCycler®480 platform (Roche Diagnostics, Switzerland). The qPCR cycles consist of an initial denaturation step at 94 °C for 5 min followed by 40 amplification cycles consisting of 94, 58, and 72 °C for 10, 30, and 30 seconds, respectively. The relative gene expression was studied using the comparative 2^-ΔΔCT^ method according to Livak and Schmittgen (2001).^83^ Dissociation curves were generated for each reaction to ensure specific amplification. Threshold values (C_T_) generated from the software tool (Roche LC 2.0) were employed to quantify relative gene expression. The sequence of primers used in the study is listed in Table S3.

#### Hemolysis assay

The hemocompatibility of the Co-deposited Ti samples was determined as per the procedure described by Patil et al. (2022).^84^ Briefly, freshly drawn sheep blood was processed by centrifugation at 500 x g to concentrate the RBCs. The RBC pellet was washed with normal saline until a clear supernatant appeared. Then concentrated RBCs were diluted with saline (1:9). 500 μL of diluted RBC suspension was added over the Ti and Co-deposited Ti samples (in triplicate) and incubated at 37°C for 1 h. After incubation, the RBC suspension was collected in a fresh microcentrifuge tube and centrifuged at 500 x g for 5 min. Following this, Drabkin’s reagent was added, and the cyanmethemoglobin formed was estimated by recording the absorbance at 540 nm using a multi-well plate reader (SynergyHT, Biotek, USA). Saline and Triton X-100 (0.1%, 50μL) were used as negative and positive controls, respectively. Percentage hemolysis was calculated using the following formula.

$$\text{Hemolysis}\;\left(\%\right)=\frac{{\text{OD}}_{\text{Test}}{-\text{OD}}_{\;\text{Negative}\;\text{control}}}{{\text{OD}}_{\text{Positive}\;\text{control}\;}{-\text{OD}\;}_{\text{Negative}\;\text{control}}}\times 100$$

### Quantification and statistical analysis

All the experiments were carried out in triplicates (n=3). All the data values are represented as mean±SD. Data analysis was performed using GraphPad Prism 5 software. One- and two-way ANOVA was applied to the data set, followed by Bonferroni post hoc test to estimate the statistical significance between the control and test groups.

## Supplemental information

Document S1. Figures S1‒S4 and Tables S1‒S3
