# Supplementary material for: Enhanced antibacterial activity and superior biocompatibility of cobalt-deposited titanium discs for possible use in implant dentistry
Source: iScience. 2024 Jan 9;27(2):108827. doi: 10.1016/j.isci.2024.108827 (PMC10831949; doi:10.1016/j.isci.2024.108827)
Supplement: Document S1. Figures S1‒S4 and Tables S1‒S3 [file mmc1.pdf]

iScience, Volume *27*

Supplemental information

Enhanced antibacterial activity and superior biocompatibility of cobalt-deposited titanium discs for possible use in implant dentistry

Vaibhav Madiwal, Bhushan Khairnar, and Jyutika Rajwade

Table S1. Elemental analysis of Ti and Co-deposited Ti surfaces using EDS, related to Figure 1.

	Elements (weight %)					
**Samples**	**C K**	**O K**	**Al K**	**Ti K**	**V K**	**Co K**
**Ti**	3.53	-	5.66	86.38	4.43	-
**Ti-Co_5**	3.7	2.14	5.29	84.17	3.82	**0.89**
**Ti-Co_{10}**	2.07	6.09	4.96	81.66	3.83	**1.39**
**Ti-Co_{15}**	3.65	8.91	5.08	76.49	3.63	**2.24**

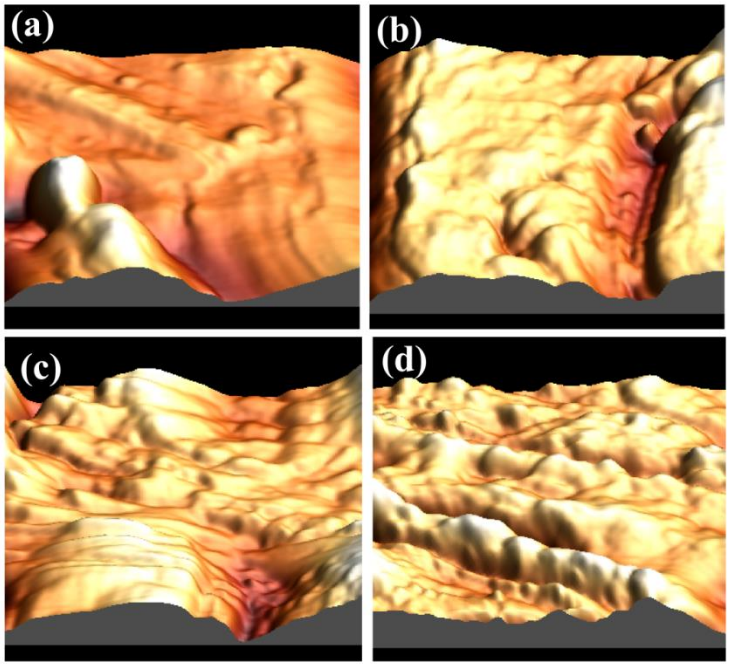

Fig. S1. 3D AFM images of Ti **(a)**, Ti-Co_5 **(b)**, Ti-Co_{10} **(c)**, and Ti-Co_{15} **(d)**

Table S2. Average elastic modulus and hardness of Ti-Co_{15} sample determined by nanoindentation, related to Figure 2

Indentation #	Avg. Modulus (Gpa)	Avg. Hardness (Gpa)
**1**	489.5	45.52
**2**	396.0	33.51
**3**	399.5	38.96
**4**	441.7	38.13
**5**	443.6	30.96
**6**	509.1	44.13
**7**	404.2	38.07
**8**	442.1	41.50
**9**	422.3	41.08
**10**	453.7	40.62
**Avg.**	**440.17**	**39.25**
**SD**	**37.42**	**4.44**

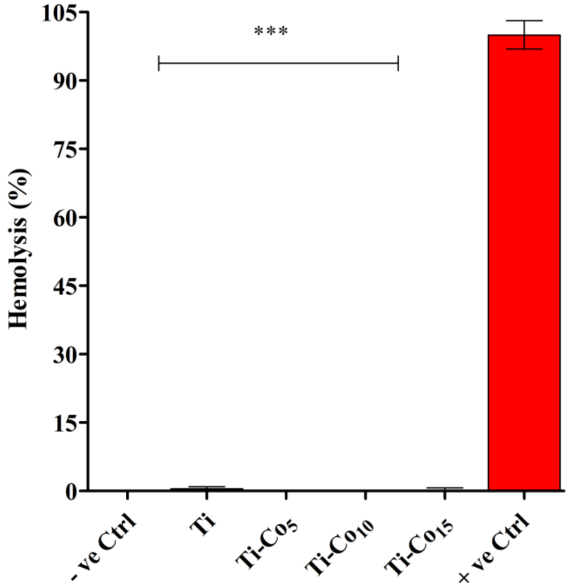

Fig. S2. Comparison of % hemolysis between Triton X-100 (+ ve control), Saline (- ve control), Ti, and Co-deposited Ti samples. *** indicates p<0.001 when compared to + ve control, related to Figure 8.

Data Item: Assessment of Co coating thickness by surface profilometry, related to STAR Methods

Coating thickness and sputter deposition rate were calculated using a surface profilometer (Fig. S3). It is a very common and easy method used to determine the metal thin film thickness. For thickness analysis, initially half of the Ti disc surface was masked with Kapton® tape (which withstands high temperature) and Co deposition was carried out for 30 min. The reason behind the higher deposition time was to get sufficiently increased step height which can be analyzed by the profilometer. After deposition, the tape was carefully removed and the samples were scanned across the surface to get the step height profile of the thin film. The deposition rate was calculated by dividing the total thickness (in nm) by deposition time and reported as nm/min.

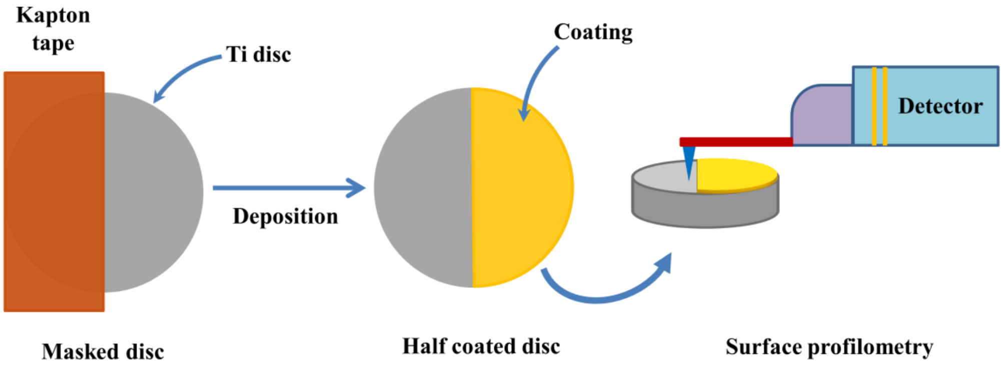

Fig. S3. Schematic of thin film thickness measurement using surface profilometry, related to STAR Methods

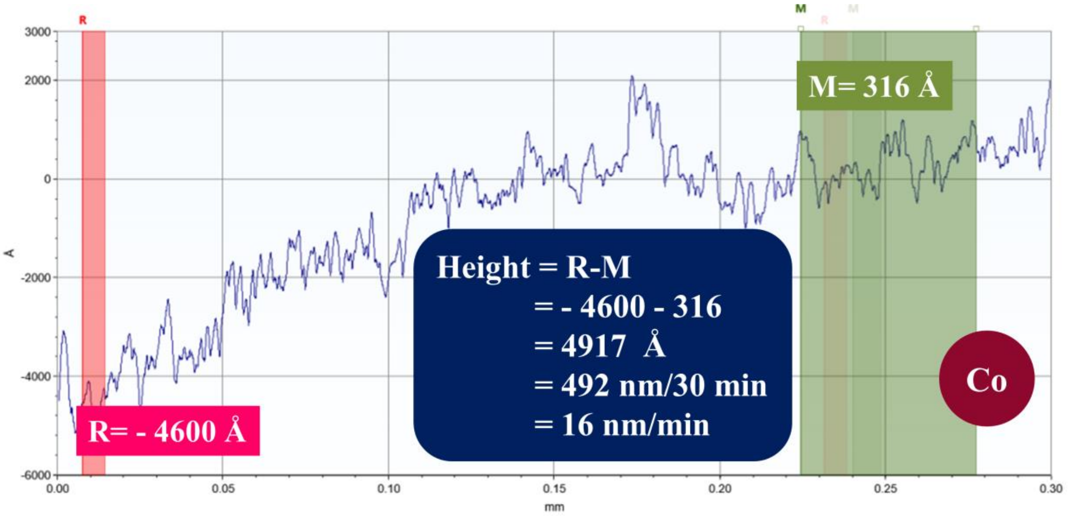

Fig. S4. Step height profile of Co-deposited Ti acquired using a profilometer, related to STAR Methods

Fig. S4 shows the step height profile of Co thin film on Ti discs after 30 min of deposition. The Co thin film thickness after 30 min deposition was 492 nm therefore Co sputter deposition rate was calculated to be 16.4 nm/min and hence Co thin film thickness on Ti-Co_5, Ti-Co_{10}, and Ti-Co_{15} would be 82, 164, and 246 nm respectively.

Table S3. Sequences of primers used in the qPCR, related to STAR Methods.

Gene	Forward seq.	Reverse seq.
GAPDH	5' TTTTGCGTCGCCAGCCGAG 3'	5’ CCAGAGTTAAAAGCAGCCCTGG 3’
ALP	5' CCCCTGAGCGTCCTGTTCT 3'	5' GGCGGCAGACTTTGGTTTC 3'
Col1α	5' AGGGCCAAGACGAAGACATC 3'	5' AGATCACGTCATCGCACAACA 3'
BMP-2	5' GGCCCTCATCAAGGGTTGGA 3'	5’AGGGTGGGCAGAAAACACGA 3’
RUNX-2	5’ CATCACTGTCCTTTGGGAGTAG 3’	5' CTTGGCCCTCCATTGTAAGA 3'
